# Metronidazole treatment of acute diarrhea in dogs: A randomized double blinded placebo‐controlled clinical trial

**DOI:** 10.1111/jvim.15664

**Published:** 2019-11-19

**Authors:** Daniel K. Langlois, Amy M. Koenigshof, Rinosh Mani

**Affiliations:** ^1^ Department of Small Animal Clinical Sciences College of Veterinary Medicine, Michigan State University East Lansing Michigan; ^2^ Veterinary Diagnostic Laboratory College of Veterinary Medicine, Michigan State University East Lansing Michigan

**Keywords:** *Clostridium perfringens*, enterotoxin, gastroenteritis, therapy

## Abstract

**Background:**

Metronidazole is commonly administered to dogs with acute diarrhea, but there is limited evidence to support this practice.

**Objective:**

To investigate the effects of metronidazole administration on dogs with acute nonspecific diarrhea.

**Animals:**

Thirty‐one dogs, including 14 test population dogs and 17 controls.

**Methods:**

Randomized controlled clinical trial. Dogs with acute diarrhea in which causation was not determined by routine fecal diagnostic testing were randomly assigned to metronidazole treatment (10‐15 mg/kg PO q12h for 7 days) or placebo. Fecal cultures and characterization of *Clostridium perfringens* isolates also were performed. Owners maintained medication and fecal scoring logs, and fecal diagnostic tests were repeated on day 7.

**Results:**

The mean ± SD time to resolution of diarrhea for test population dogs (2.1 ± 1.6 days) was less than that for controls (3.6 ± 2.1 days, *P* = .04). Potential relationships of *C. perfringens* with acute diarrhea pathogenesis were not investigated, but only 3 of 13 (23.1%) test population dogs had persistent *C. perfringens* carriage at day 7, which was less than the 11 of 14 (78.6%) controls with persistent growth (*P* = .007).

**Conclusions and Clinical Importance:**

Our results suggest that metronidazole treatment can shorten duration of diarrhea and decrease fecal culture detection of *C. perfringens* in some dogs with acute nonspecific diarrhea. Additional studies are needed to assess the benefits and risks of routine use of metronidazole for this purpose because most dogs achieve resolution of diarrhea within several days regardless of treatment.

**Abbreviations**
MSU‐VDL: Michigan State University Veterinary Diagnostic Laboratory
MSU‐VMC: Michigan State University Veterinary Medical Center

## 1 INTRODUCTION

Acute diarrhea, which is defined as a short‐term increase in the liquidity of feces, is a common reason for non‐wellness–related veterinary evaluations in dogs.^1^, ^2^, ^3^, ^4^ Most cases are mild and self‐limiting, but owners frequently seek veterinary care because of concern for their pet's well‐being in conjunction with the difficulties associated with managing a dog with diarrhea.^1^, ^2^, ^5^ The causes of acute diarrhea are varied. Infectious organisms and parasites account for some cases, but routine diagnostic testing often fails to identify a specific cause.^6^ Extensive diagnostic tests are seldom performed because the yield is low, especially if the patient is clinically stable.^7^, ^8^ Recent environmental changes, stress, dietary indiscretion, rapid diet changes, and medication administration are commonly associated with nonspecific cases of acute diarrhea in dogs.^1^, ^2^ Alterations in the normal microbiota also could be involved in the pathogenesis.^9^ Although acute diarrhea is typically self‐limiting, treatment is commonly administered in attempt to lessen the severity or duration of diarrhea. Probiotics, antibiotics, and dietary modifications, alone or in combination, often are used for this purpose.^10^, ^11^, ^12^, ^13^

Many veterinarians prescribe metronidazole, a nitroimidazole antibiotic, for the treatment of acute diarrhea in dogs.^1^, ^10^, ^13^ It has a broad spectrum of activity against anaerobic bacteria including potential enteric pathogens such as *Clostridium perfringens*, and antiprotozoal activity is observed at higher dosages.^14^, ^15^, ^16^ Beyond its antimicrobial activity, metronidazole possesses immunomodulatory and anti‐inflammatory properties that further add to its appeal for treating intestinal disorders.^17^ However, controlled studies of metronidazole treatment of acute diarrhea are limited, and it is unknown if metronidazole alters the clinical course of disease.^10^, ^18^ The commonplace usage of antibiotics for treating a self‐limiting condition raises concerns regarding appropriate antimicrobial stewardship because this practice could promote bacterial resistance.^19^ Metronidazole also has been associated with an increased risk of cancer and other adverse sequelae in humans.^20^ Considering these concerns, it is important to establish whether or not metronidazole is of clinical benefit for the routine treatment of acute diarrhea. The primary objectives of this randomized controlled clinical trial were to evaluate the effects of metronidazole treatment on duration of diarrhea in dogs with acute nonspecific diarrhea. We also sought to assess treatment effects on *C. perfringens* as determined by fecal cultures and molecular techniques. We hypothesized that metronidazole treatment would shorten the duration of diarrhea as compared to placebo. We further hypothesized that metronidazole treatment would decrease fecal culture detection of *C. perfringens*.

## 2 MATERIALS AND METHODS

### 2.1 Animals

Dogs evaluated at the Michigan State University Veterinary Medical Center (MSU‐VMC) for acute diarrhea, with or without concurrent vomiting, were screened for participation in a randomized, placebo‐controlled, double‐blinded clinical trial. A sample size calculation using hospital data and assuming probability (power) of .8 and *α* of .05 suggested studying 15 experimental subjects and 15 control subjects to be able to detect a 1.5 day difference in duration of diarrhea. Study investigators sought to enroll up to 20 dogs in each treatment arm to account for possible exclusions. Inclusion criteria included age >6 months, body weight between 4 and 50 kg, and active diarrhea <7 days in duration. Dogs were required to be up‐to‐date on core vaccinations.^21^ Dogs receiving probiotics, antibiotics, or anti‐inflammatory treatments in the preceding 30 days were excluded as were pregnant or nursing dogs. Dogs with moderate to severe abdominal pain, complete anorexia, or moderate to severe dehydration (estimated at >8%) were excluded from participation. Eligible dogs then underwent a series of fecal diagnostic tests as described below. Dogs that did not have evidence of gastrointestinal parasitism, *Giardia* spp. infection, or parvoviral enteritis were considered to have acute nonspecific diarrhea and enrolled in the clinical trial. Additional diagnostic testing was performed at the discretion of the attending clinician.

### 2.2 Fecal diagnostic testing

Fresh fecal samples were obtained from all dogs at baseline and at day 7 evaluation. Antigen testing for *Giardia* spp. (SNAP Giardia Test, IDEXX Laboratories, Westbrook, Maine) and canine parvovirus (SNAP Parvo Test, IDEXX Laboratories) was performed using commercially available point‐of‐care ELISA tests. A standard centrifugal fecal flotation was performed at the Michigan State University Veterinary Diagnostic Laboratory (MSU‐VDL), which is an American Association of Veterinary Laboratory Diagnosticians‐accredited laboratory. Aerobic and anaerobic/*Clostridial* spp. fecal microbial cultures also were performed at the MSU‐VDL. In brief, fecal samples for aerobic bacterial culture were plated on trypticase soy agar with 5% sheep blood (TSAB), MacConkey agar, and Columbia colistin‐nalidixic acid agar with 5% sheep blood (CNA). Samples for *Clostridial* spp. culture were plated on phenylethyl alcohol agar with 5% sheep blood and maintained under anaerobic conditions. *Salmonella* spp. enrichment culture using tetrathionate hajna broth and subsequent plating on xylose‐lysine‐tergitol and brilliant green plates also were performed. Isolated *C. perfringens* was typed by toxin‐gene PCR. The bacterial DNA was extracted by boiling bacteria in sterile water on a heat block for 20 minutes. A multiplex PCR was performed using specific primers for multiple toxin genes which included the genes for *C. perfringens* enterotoxin (*cpe*) and beta 2 toxin (*cpb2*).^22^, ^23^ The PCR products, including negative and positive template controls, were analyzed on a 1.5% agarose gel.

### 2.3 Drug compounding

Pharmaceutical grade metronidazole (Unichem Laboratories Ltd, Mumbai, India) was compounded into gelatin capsules containing 25, 50, 100, 200, and 300 mg of active product with microcrystalline cellulose used as an excipient. These capsule sizes, administered alone or in combination, would enable administration of the target metronidazole dosage (10‐15 mg/kg PO q12h for 7 days) for the wide range of body weights included in the study. Placebo capsules of identical size and appearance were prepared with microcrystalline cellulose as the only compound. Capsules were stored in an automated drug dispensary (MedFlex 2000, Cubex LLC, Phoenix, Arizona) that was maintained by the MSU‐VMC pharmacist and pharmacy technician. The pharmacist and pharmacy technician were the only personnel not blinded to treatment group.

### 2.4 Experimental protocol

Dogs were assigned to treatment groups in double‐blinded fashion using a computer generated randomization log that was created by the pharmacist. At the time of enrollment, the attending clinician retrieved 16 doses of drug or placebo per dog from the automated dispensary which was programmed to dispense capsules based on randomization order. Owners were instructed to administer the prescribed doses at approximately 12 hour intervals for 7 days beginning on the day of enrollment. The additional doses were provided in case of administration difficulties or lost capsules. Owners maintained medication logs (Supplementary File S1) and recorded the time of drug administration. Owners also maintained fecal scoring logs (Supplementary File S2) using the Bristol scoring system (Supplementary File S3), and a laminated copy of the Bristol fecal chart was provided to owners to aid in fecal scoring. This scoring system has not been validated in dogs, but it has been validated and utilized extensively in humans and it shares many similarities with other non‐validated scoring scales that have been used in studies of diarrheic dogs.^5^, ^18^, ^24^, ^25^, ^26^, ^27^ Briefly, fecal scores could range from 1 to 7, with scores of ≤4 considered to be non‐diarrheic.^25^, ^26^ The time and score of each observed defecation, including a pretreatment baseline assessment, were recorded. Owners also were instructed to withhold food from their dogs for the initial 12 hours after enrollment and gradually resume feeding of the normal diet over the subsequent 12‐24 hours. Dogs that had vomited before presentation or those deemed to be nauseated were treated with a single 1 mg/kg SC dose of maropitant citrate (Cerenia, Zoetis, Kalamazoo, Michigan) at the discretion of the attending clinician. Administration of crystalloid fluids also was permitted. Additional treatment with antacids, antidiarrheals, or any newly prescribed medications was not permitted for the duration of the study unless the dog's clinical condition deteriorated and warranted treatment. In such cases, dogs were removed from the clinical trial.

Dogs were returned for repeat evaluation and fecal diagnostic testing on day 7, and treatment group was unmasked. Medication and fecal scoring logs as well as unused drug were collected from owners. The study was concluded in all test population dogs as well as control population dogs in which diarrhea had resolved. Further veterinary care of these patients was independent of study participation. Control population dogs with persistent diarrhea at the day 7 evaluation then were treated with a 7‐day course of metronidazole in open label fashion. Owners of these dogs continued to maintain fecal scoring and medication logs. These dogs returned for a final evaluation and fecal diagnostic testing on day 14. Monitoring logs and unused drug were collected, and the study was concluded at that time.

### 2.5 Data and Statistical analysis

Data sets were assessed for normality by Shapiro‐Wilk testing and inspection of normal probability plots. Normally distributed data were reported as means ± SD whereas data not approximating normal distributions were reported as medians and interquartile ranges (IQR). Resolution of diarrhea was defined as the time at which the first of 2 consecutive fecal scores ≤4 was recorded. Time 0 was the time at which initial drug or placebo administration occurred. Dogs with persistent diarrhea after the initial week of treatment were considered to have resolution on day 7 for statistical purposes. A multivariable linear regression analysis was performed with the primary study outcome of duration of diarrhea. In addition to treatment group, the variables of age, sex, weight, baseline fecal score, use of fluid therapy, and use of maropitant citrate were considered in the regression model. Variables with *P* ≥ .20 were removed from the model, but all deleted variables were individually added back to the final model to assess for significance. Potential differences in the proportion of dogs with fecal cultures positive for *C. perfringens* growth were compared using Fisher exact testing. Statistical analyses were performed using commercially available software (SAS, version 9.3, SAS Institute Inc, Cary, North Carolina), and for all analyses, values of *P* ≤ .05 were considered significant.

## 3 RESULTS

### 3.1 Dogs

Forty‐eight dogs were screened for study enrollment, 14 of which were excluded from participation because of gastrointestinal parasitism (n = 10), inability to obtain sufficient feces for fecal testing (n = 2), normal fecal score before initiation of study drug (n = 1), or ultrasonographic evidence of acute pancreatitis (n = 1). One dog was removed from the study shortly after enrollment because of severe vomiting that warranted additional antiemetic treatment. This dog was in the test population, had received only 1 dose of study drug, and was removed <24 hours after enrollment. Serious protocol deviations, including failure to score feces and return for the day 7 recheck evaluation (n = 1) and failure to score feces and adhere to dietary guidelines (n = 1), resulted in exclusion of 2 additional test population dogs. Both owners reported resolution of diarrhea, but these dogs were not included in analyses because the exact time at which resolution occurred was unknown.

The 31 dogs meeting all inclusion criteria and completing the study according to protocol guidelines included 14 test population dogs and 17 controls. Demographics and baseline characteristics of the test and control population dogs were similar and are summarized in Table 1. Seven of 14 (50%) test population dogs and 11 of 17 (64.7%) control dogs had hematochezia noted by owners or attending clinicians which was not different between groups (*P* = .48). Eight of 14 (57.1%) test population dogs received fluid therapy which included SC (n = 4) or IV (n = 4) administration. Seven of 17 (41.2%) control population dogs received fluid therapy which included SC (n = 2) or IV (n = 5) administration. For dogs receiving IV fluid therapy, the duration of treatment was ≤1 day. The proportion of dogs receiving fluid therapy in each population was not different (*P* = .48). Six of 14 (42.9%) test population dogs and 6 of 17 (35.3%) control dogs were treated with maropitant citrate for concurrent nausea and vomiting, which also was not different between populations (*P* = .72). Eighteen dogs, including 8 test population dogs and 10 controls, underwent routine serum or plasma biochemical evaluations. Evidence of renal, hepatic, or metabolic disease was not present in any of these dogs. No dogs in either population had growth of *Salmonella* spp., *Camplylobacter* spp., or *Clostridioides difficile* on fecal cultures at any time during the study. Fecal flotations for parasite ova and antigen testing for *Giardia* spp. and canine parvovirus were negative at baseline and day 7 in all study dogs. No dogs developed treatment‐related adverse effects or evidence of metronidazole‐induced neurotoxicity.^28^

**Table 1:** Characteristics of the 31 dogs participating in a randomized controlled clinical trial in which the effects of metronidazole treatment for acute diarrhea were evaluated

Variable	Test, n = 14	Control, n = 17	*P* value
Age (years)	4.1 ± 3.4	4.9 ± 3.6	.53
Sex (male/female)	7/7	8/9	.99
Weight (kg)	17.9 ± 9.4	20.9 ± 16.3	.55
Fecal score	7 (7–7)	7 (7–7)	.23

*Notes*: Data are shown as mean ± SD, absolute number, or median (interquartile range), in the specified groups. Test population dogs received metronidazole (10‐15 mg/kg PO q12h for 7 days), whereas the control dogs received placebo. All characteristics reflect baseline values immediately before study enrollment. The fecal score was based on the Bristol fecal chart with possible scores ranging from 0 to 7.^25^, ^26^, ^27^ Scores ≤4 are considered non‐diarrheic. All test population dogs had a baseline fecal score of 7, whereas 14 of 17 (82.4%) control dogs had a baseline fecal score of 7. The remaining 3 control dogs had a baseline score of 6. The *P* value reflects univariate comparisons of each variable between test and control dogs. Note, none of the baseline characteristics were different between populations.

### 3.2 Treatment effects

**Figure 1:**
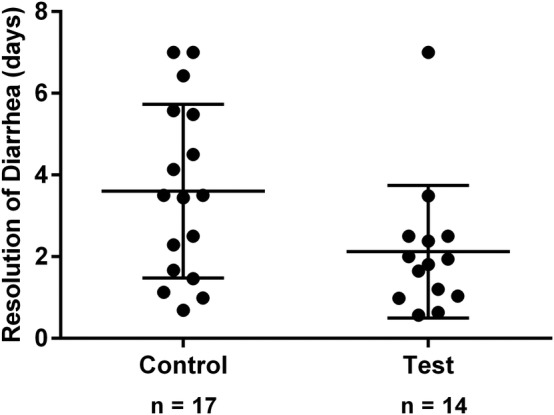
Scatter plot depicting the duration of time (days) until resolution of diarrhea in the 31 dogs with acute diarrhea that were randomly assigned to receive treatment with metronidazole (10‐15 mg/kg PO q12h for 7 days) or placebo in double blinded fashion. The duration of time until resolution of diarrhea was defined as the time at which the first of 2 consecutive fecal scores ≤4 were recorded in relation to the initiation of treatment or placebo. The central and outer horizontal lines within each scatter represent the mean and SD, respectively. The time of 2.1 ± 1.6 days in test population dogs was less than the time of 3.6 ± 2.1 days in controls (*P* = .04)

Age (*P* = .25), sex (*P* = .85), weight (*P* = .76), maropitant citrate treatment (*P* = .74), baseline fecal score (*P* = .39), and fluid therapy (*P* = .20) were not associated with duration of time until resolution of diarrhea in the multivariable analysis, but treatment group was a significant factor. Resolution of diarrhea was achieved 1.5 days faster in test population dogs than in control dogs (Figure 1; *P* = .04). All test population dogs had resolution of diarrhea in <4 days with the exception of 1 dog in which fecal scores ≤4 were not observed until day 7. Seven control dogs had diarrhea lasting >4 days, including 2 control dogs that had persistent diarrhea at day 7. Both control dogs with persistent diarrhea then were treated with metronidazole in open‐label fashion, and diarrhea resolved 1.1 and 4.9 days after treatment was initiated. No dog in either populations had a relapse in clinical signs through day 21, but long‐term monitoring was not performed.

Overall, 27 of 31 (87.1%) dogs had positive fecal cultures for *C. perfringens* on day 0 including 13 of 14 (92.9%) test population dogs and 14 of 17 (82.4%) control dogs. The proportion of dogs with baseline fecal culture growth of *C. perfringens* was not different between test and control populations (*P* = .61). Molecular typing determined that all isolates were type A strains. Within the test population, 4 samples were positive for *cpb2*, 1 sample was positive for *cpe*, and 1 sample was positive for both genes. Within the control population, 3 samples were positive for *cpb2*, and 3 samples were positive for *cpe*. Hematochezia was not associated with the detection of toxin encoding genes because 7 of 18 (38.9%) dogs with hematochezia and 5 of 13 (38.5%) dogs without hematochezia had positive toxin gene detection (*P* = .99). Only 3 of 13 (23.1%) test population dogs remained positive for fecal culture growth of *C. perfringens* on day 7 which is less than the 11 of 14 (78.6%) control dogs that remained positive for *C. perfringens* growth on day 7 (*P* = .007). Of the 2 control dogs that had persistent diarrhea on day 7, 1 dog was positive for *C. perfringens* growth and *cpe* on both day 0 and day 7. A fecal sample from this dog was negative for *C. perfringens* growth on day 14, which was after 1 week of metronidazole treatment.

## 4 DISCUSSION

Our results suggest that metronidazole treatment can shorten the duration of diarrhea in some dogs with acute nonspecific diarrhea. Overall, the duration of diarrhea was decreased by approximately 1.5 days in metronidazole‐treated dogs as compared to placebo‐treated dogs (*P* = .04). This statistical difference could be of clinical relevance because a 1.5 day decrease in clinical signs would be appealing for clients challenged with managing a diarrheic pet. However, the results of our study do not establish whether or not metronidazole should be utilized as a first line drug for acute diarrhea because many additional factors must be considered. Most dogs (88.2%) have resolution of diarrhea within 1 week even in the absence of treatment. Also, metronidazole is not approved by the Food and Drug Administration for veterinary purposes despite its commonplace usage in companion animal medicine.^29^ Acute diarrhea is a common reason owners seek veterinary care, and widespread antimicrobial treatment could impact both veterinary and human health by promoting bacterial resistance.^19^ Additionally, evidence suggests that metronidazole treatment dramatically alters the intestinal microbiome of normal dogs, and some of these changes persist for an indefinite time period.^30^ Dietary modifications or probiotic treatment might yield clinically advantageous effects comparable to those of metronidazole and preclude the need for antimicrobial treatment.^5^, ^10^, ^31^, ^32^ These and other important issues require further consideration, but they were beyond the scope of our study.

Management strategies for acute diarrhea in dogs have garnered considerable attention in recent years.^1^, ^2^, ^3^, ^4^, ^5^, ^10^, ^11^, ^12^, ^13^ Probiotics have been studied extensively, but with inconsistent results. Various formulations have been documented to decrease the duration of acute diarrhea by 0.6,^5^ 0.9,^12^ 1,^32^ and 2.7 days^31^ as compared to placebo‐treated dogs. Other studies have shown no treatment effects, and a recent systematic review suggested that the overall clinical benefit of probiotic therapy is potentially unimportant.^18^, ^33^ Short‐term dietary modifications often are recommended in dogs with acute diarrhea, but strong evidence for this practice is lacking.^1^, ^2^ Studies of metronidazole treatment of acute diarrhea in dogs are limited.^10^, ^18^ Concurrent treatments frequently are administered in clinical studies of acute diarrhea, different fecal scoring scales often are utilized, and the definition of diarrhea resolution is not uniform.^10^, ^18^, ^32^ Consequently, interpretation and application of results are challenging. A previous randomized controlled clinical trial failed to document a benefit of metronidazole treatment as compared to placebo in dogs with acute nonspecific diarrhea.^18^ The reasons for conflicting results are unclear. The average durations of time until diarrhea resolution in both metronidazole (4.6 days) and placebo (4.8 days) treated dogs from the aforementioned study were longer than the average durations of time reported in our study, which suggests that the study populations may not be similar. Different designs and methodologies could have contributed to discordant study results. In the previous trial, some dogs did not have fecal flotations performed, clinicians were permitted to treat dogs with an anthelmintic at their discretion, and owners were not required to document daily fecal scores.^18^ We also utilized a different fecal scoring system, which has not been used routinely in studies of dogs despite widespread use in clinical trials of humans.^24^, ^25^, ^26^ The Bristol fecal chart appeared to perform adequately because all dogs had fecal scores of 6 or 7 at baseline, and all dogs had scores ≤4 at the time of study termination. Regardless, continued clinical research is needed to better characterize treatment approaches for dogs with acute diarrhea.

*Clostridium perfringens* is likely to be involved in the pathogenesis of various diarrheal syndromes in dogs.^16^, ^32^, ^34^ Both *C. perfringens* enterotoxin and *cpe* are more commonly detected in dogs with diarrhea than in non‐diarrheic dogs.^34^, ^35^ Similarly, the *cpb2* gene recently was identified in a larger number of *C. perfringens* isolates from diarrheic dogs as compared to non‐diarrheic dogs.^36^ Additional *C. perfringens* genes and associated toxins also seem to be involved in specific forms of disease such as acute hemorrhagic diarrhea syndrome.^16^ Although *C. perfringens* and its toxins often are associated with acute diarrhea, it is still unclear if this bacteria is a cause or consequence of the intestinal disease.^34^, ^35^, ^37^ We did not evaluate *C. perfringens* in a healthy population of dogs, but previous studies have shown that isolation rates are similar in diarrheic and non‐diarrheic dogs, and the *C. perfringens* toxins associated with diarrhea also can be identified in healthy, non‐diarrheic dogs.^34^, ^35^, ^36^, ^37^, ^38^ Further complicating matters, the presence of toxin‐encoding genes does not always correlate to the presence of actual toxins.^34^, ^35^ These findings pose obvious diagnostic challenges for individual cases. Our study was not designed to establish a relationship between *C. perfringens* and acute diarrhea, but we still thought it was important to consider the effects of metronidazole treatment on *C. perfringens* carriage given the potential associations with acute diarrhea that have been observed in other studies. The frequency at which *C. perfringens* was detected by fecal culture in our population of diarrheic dogs (87.1%) is similar to previous reports, as is the frequency of *cpe* and *cpb2* identification.^34^, ^35^, ^39^ More importantly, metronidazole treatment resulted in negative fecal cultures for *C. perfringens* in approximately 80% of dogs with positive cultures at baseline. This finding is in contrast to the nearly 80% of control dogs that remained positive for *C. perfringens* growth on day 7. These findings are in agreement with previous reports that suggest metronidazole is an effective treatment for most *C. perfringens* isolates.^14^, ^40^

Our inclusion criteria, which selected clinically stable dogs that were still eating and drinking, may have excluded dogs with nongastrointestinal or metabolic diseases. This conclusion is further supported by the normal biochemical evaluations that were performed in 18 dogs. However, comprehensive diagnostic testing was not performed in many dogs of the present study, and heterogeneous etiologies for acute diarrhea could have been represented in the study population. Although this could be perceived as a limitation, extensive diagnostic testing such as abdominal ultrasound examination and various molecular‐based diagnostic fecal panels are of questionable utility in clinically stable dogs with acute diarrhea.^7^, ^8^, ^34^ Each dog in our study did undergo repeated fecal diagnostic tests to ensure that gastrointestinal parasitism and parvovirus infection were not present, and baseline characteristics were similar between test and control populations. Also, none of the potential confounding factors considered in the multivariable analysis were found to be related to the duration of diarrhea. Another limitation is that long‐term monitoring was not performed as part of this study, and acute diarrhea might be an early manifestation of a more chronic disease process. The sample size of 31 is small for a randomized controlled clinical trial, and different results might be obtained with a larger population. Severe protocol deviations led to exclusion of 2 dogs, which was not unanticipated given the requirements for owners to document numerous fecal scores and return for a repeat clinical evaluation. However, we did not expect 22% of dogs screened for enrollment to be affected by gastrointestinal parasitism given that our study population received routine veterinary care. Lower parasitism prevalence rates have been reported in some studies, but these results reflect convenience samples from both healthy and diarrheic dogs.^41^, ^42^ Regional prevalence studies are not available for the State of Michigan, but a similar prevalence rate has been reported in diarrheic dogs in other regions.^43^ Although not an intended study aim, the high rate of gastrointestinal parasitism underscores the importance of fecal flotations in dogs with acute diarrhea.

In summary, metronidazole treatment modestly decreased the duration of diarrhea in this population of dogs with acute nonspecific diarrhea. Metronidazole treatment also decreased *C. perfringens* carriage although the relationship of *C. perfringens* with acute diarrhea was not investigated. The usage of metronidazole should be carefully considered in cases of acute diarrhea because our results do not preclude the possibility that other treatments could be similarly or even more effective. The impact of widespread antimicrobial usage for a condition that is frequently self‐limiting also needs to be considered. Future studies are needed to substantiate our results and address these concerns before metronidazole treatment of acute diarrhea in dogs can be categorically recommended.

## CONFLICT OF INTEREST DECLARATION

Dr. Langlois serves on a scientific advisory board for Zomedica, Inc. The authors declare no additional conflicts of interest.

## OFF‐LABEL ANTIMICROBIAL DECLARATION

Metronidazole is not approved for use in dogs in the U.S.

## INSTITUTIONAL ANIMAL CARE AND USE COMMITTEE APPROVAL

This study was approved by the Michigan State University IACUC, and informed consent was obtained from all owners.

## HUMAN ETHICS APPROVAL DECLARATION

Authors declare human ethics approval was not needed for this study.

## Supporting information


**Appendix S1**: Supporting Information

**Appendix S2**: Supporting Information

**Appendix S3**: Supporting Information
