# Supplementary material for: Metronidazole treatment of acute diarrhea in dogs: A randomized double blinded placebo‐controlled clinical trial
Source: J Vet Intern Med. 2019 Nov 19;34(1):98–104. doi: 10.1111/jvim.15664 (PMC6979100; doi:10.1111/jvim.15664)
Supplement: Supplementary file 1 — Appendix S1: Supporting Information [file JVIM-34-98-s001.pdf]

Appendix 1

Drug / dose ______________________________

	Medication					Adverse Events *							
			Dose given			worsening diarrhea		anorexia		lethargy		Other Effect	
	Date	Dose	yes	no	Time	yes	no	yes	no	yes	no	yes	no
WEEK 1		AM											
		PM											
		AM											
		PM											
		AM											
		PM											
		AM											
		PM											
		AM											
		PM											
		AM											
		PM											
		AM											
		PM											

*Adverse Event - If any adverse events were noted above, please elaborate, in detail, below.
