# Supplementary material for: Metronidazole treatment of acute diarrhea in dogs: A randomized double blinded placebo‐controlled clinical trial
Source: J Vet Intern Med. 2019 Nov 19;34(1):98–104. doi: 10.1111/jvim.15664 (PMC6979100; doi:10.1111/jvim.15664)
Supplement: Supplementary file 2 — Appendix S2: Supporting Information [file JVIM-34-98-s002.pdf]

STOOL GRADING FORM

Animal ID:

____
(Case ID)

Document date, time, grade score and any applicable comments.

Date	Time<br>Circle am or pm	Grade<br>Indicate 1-7 from Bristol Stool Chart	Comments
	am<br>pm		
	am<br>pm		
	am<br>pm		
	am<br>pm		
	am<br>pm		
	am<br>pm		
	am<br>pm		
	am<br>pm		
	am<br>pm		
	am<br>pm		
	am<br>pm		
	am<br>pm		
	am<br>pm		
	am<br>pm		
	am<br>pm		
	am<br>pm		
	am<br>pm		
	am<br>pm		
	am<br>pm		
	am<br>pm		

Date	Time<br>Circle am or pm	Grade<br>Indicate 1-7 from Bristol Stool Chart	Comments
	am<br>pm		
	am<br>pm		
	am<br>pm		
	am<br>pm		
	am<br>pm		
	am<br>pm		
	am<br>pm		
	am<br>pm		
	am<br>pm		
	am<br>pm		
	am<br>pm		
	am<br>pm		
	am<br>pm		
	am<br>pm		
	am<br>pm		
	am<br>pm		
	am<br>pm		
	am<br>pm		
	am<br>pm		
	am<br>pm		
	am<br>pm		
	am<br>pm		
	am<br>pm		
	am<br>pm		
	am<br>pm		
	am<br>pm		
	am<br>pm		

Date	Time<br>Circle am or pm	Grade<br>Indicate 1-7 from Bristol Stool Chart	Comments
	am<br>pm		
	am<br>pm		
	am<br>pm		
	am<br>pm		
	am<br>pm		
	am<br>pm		
	am<br>pm		
	am<br>pm		
	am<br>pm		
	am<br>pm		
	am<br>pm		
	am<br>pm		
	am<br>pm		
	am<br>pm		
	am<br>pm		
	am<br>pm		
	am<br>pm		
	am<br>pm		
	am<br>pm		
	am<br>pm		
	am<br>pm		
	am<br>pm		
	am<br>pm		
	am<br>pm		
	am<br>pm		
	am<br>pm		
