# Supplementary material for: Metronidazole treatment of acute diarrhea in dogs: A randomized double blinded placebo‐controlled clinical trial
Source: J Vet Intern Med. 2019 Nov 19;34(1):98–104. doi: 10.1111/jvim.15664 (PMC6979100; doi:10.1111/jvim.15664)
Supplement: Supplementary file 3 — Appendix S3: Supporting Information [file JVIM-34-98-s003.pdf]

Bristol Stool Chart

Type 1	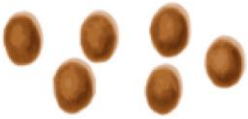	• Separate hard lumps, like nuts<br>• Often expelled as individual pellets<br>• Hard to pass<br>• No residue left on ground when picked up
Type 2	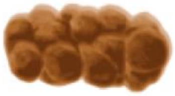	• Sausage-shaped but lumpy<br>• Firm but not hard<br>• Should be pliable<br>• Little or no residue left on ground when picked up
Type 3	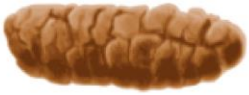	• Like a sausage but with cracks on its surface<br>• Moist surface<br>• Leaves residue but holds form when picked up
Type 4	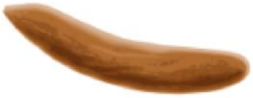	• Like a sausage or snake, smooth and soft<br>• Leaves residue and loses form when picked up
Type 5	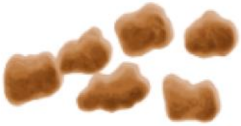	• Soft blobs or piles with clear-cut edges<br>• Easy to pass<br>• Leaves residue and loses form when picked up
Type 6	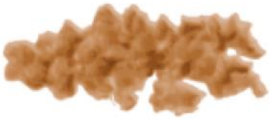	• Fluffy pieces with ragged edges<br>• A mushy stool<br>• No defined shape (piles or spots)<br>• Leaves residue when picked up
Type 7	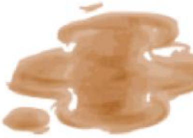	• Watery with no solid pieces<br>• **Entirely liquid**
